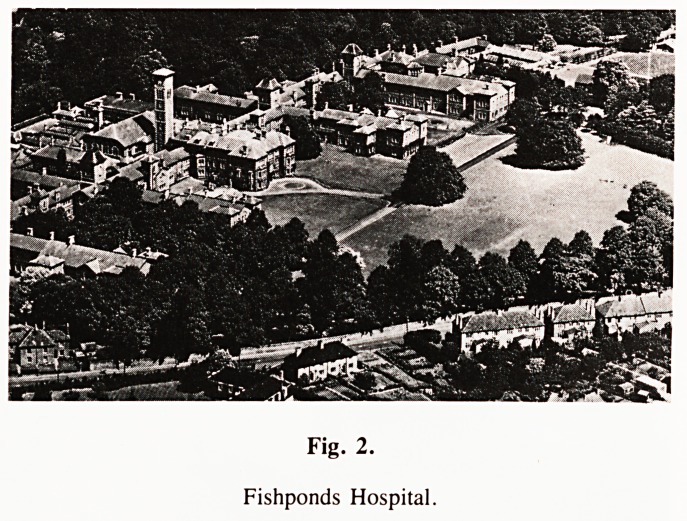# Lunacy Bristol Fashion

**Published:** 1991-12

**Authors:** Donal Early


### Lunacy Bristol Fashion

### Bristol Medico-Historical Society, 16th October 1989

**Donal Early, FRCPsych, DPH**

As elsewhere there are two main historical strands in the development of psychiatric services in Bristol. There were no separate statutory services provided until the 19th Century, the care of the mentally ill being the responsibility of the Poor Law. Where in need they shared the common lot of other paupers in the workhouses and the work-house infirmaries. Private services were provided for the well to do. During the 17th and 18th Centuries the provision of private services was a growth industry, as unfortunately it has again become in the latter part of the 20th Century. The private mad houses were frequently run by charlatans and were regarded with grave suspicion. This public unease became evident towards the end of the 18th Century when the treatment of King George III gave rise to public criticism. George III had his first attack of "mania" in 1788. Public awareness was further stimulated by the appointment by the Paris Commune in 1793 of a physician (Pinel) to the Bicetre Hospital where he immediately introduced liberal reforms. Under the influence of the Society of Friends the retreat in York was founded in 1792 under the superintendency of William Tuke.

There were many private mad houses in the vicinity of Bristol. The Lunatic Asylum in Fishponds was opened by Joseph Mason in 1740 and continued to function until closed following an official enquiry in 1859. Richard Henderson ran a house in Cleeve which was glowingly praised by John Wesley; the management of this house was taken over by Edward Long Fox who built and opened Brislington House, in 1804. This and other local private hospitals survived the National Health Service for example Winterbourne House, Bailbrook House, Bath, and Barnwood House, Gloucester.

The statutory provision of accommodation for lunatics was imposed on local authorities by the Asylum's Act of 1808. As usual with such legislation little happened until the requirement became mandatory by the passing of the Lunatics Act of 1845. Even after this Bristol refused to act in spite of "almost unqualifed censure" by the Commissioners in Lunacy in 1844. Bristol was enabled to avoid responsibility by the existence of lunatic beds in St. Peter's General Hospital.

In its day St. Peter's Hospital was well ahead of its time. John Cary, "a merchant of Bristol" proposed in the Bristol Poor Act 1696 that responsibility for the destitute poor became the responsibility of a combination of the parishes of the city with a uniform rate levied in each parish to meet the cost of relief. Thus, the able bodied would be compelled to work, the infirm would be cared for, the young would be adequately trained, expenditure would be saved and litigation between parishes would disappear. The Board of Guardians of the Corporation of the Poor held its meetings in St. Peter's from 20th October 1696 until the building was destroyed by enemy bombing in 1940.

The Bristol Poor Act of 1696 pre-dated similar national legislation by more than 100 years and St. Peter's was unique in that it undertook the treatment of pauper lunatics — probably the first provincial hospital to do so. It provided a regime of treatment liberal by the then standards. Over the years conditions deteriorated until it was described in the mid 19th Century as "totally unfit to be an asylum". Nevertheless by virtue of the existence of asylum wards in St. Peter's the city fathers were able to evade their duties under the Lunatic's Act 1845 until cumulative pressures compelled them to build the Bristol Lunatic Asylum which received 50 males and 65 female patients from St. Peter's Hospital on February 27th 1861

**Fig. 1.**

St. Peter's Hospital.

The development of the asylum in the early days was typical of its time with overcrowding rapidly overtaking development. Extensions took place over the years; by 1882 there were plans to extend to 750 patients and by 1927 there were over 1,000 beds.

Nurse training was introduced more than 100 years ago. The Royal Medico Psychological Association which was founded in 1841 issued the Red Handbook for nurses in 1885 and introduced a certificate of nursing in 1890; all nurses in charge of patients had this qualification within 5 years. Dissatisfaction with terms and conditions is not a 20th Century phenomenon. There was a strike in 1890 and all the staff were removed except the Chief Male Nurse. It is interesting to note that in 100 years the ratio of staff to patients has gone from about 14 or 15 patients to one staff to 15 staff to 8 places (in St. Maur, Newton Abbot 1989).

**Fig. 2.**

Fishponds Hospital.

A recital of events year by year becomes a tedious litany. International and national events played their part in the history of the hospital. During the first world war 50% of the staff were called up and the hospital was taken over by the military and run as a military hospital under the name of the Beaufort War Hospital. Soon after it was reopened it was named the Bristol Mental Hospital and remained so until 1959 when the Mental Health Act of that year led to the mundane name of "Glenside Hospital".

The inter-relationship between psychiatry and general medicine was maintained with the re-establishment of an Out Patient Department at the Bristol Royal Infirmary in 1922 followed soon afterwards by the equipment of a research department in the Mental Hospital. Doctor Barton White the Medical Superintendent was appointed Lecturer in Mental Disorders at the University of Bristol in 1925 but it was not until 1967 that a Chair of mental health was established and Professor Russell Davis was appointed.

Development was steady, visiting specialists were appointed in surgery, midwifery and gynaecology, medicine and opthalmology in the twenties when occupational therapy was also started. Occupational therapy as a profession is associated with the name of Dr. Elizabeth Casson the Bristol pioneer who introduced her ideas in her Centre on the Downs.

By the late twenties it was agreed that overcrowding had become unbearable and in 1932 the City Council allocated £195,000 towards the building of a new hospital at Barrow. This hospital officially opened in May 1939 but was taken over by the Royal Navy the following September at the outbreak of war.

In April 1939 the Burden Neurological Institute was opened. The Visiting Committee of the mental hospital agreed to assist financially in the running of the Burden which co-operated in the investigation of mental diseases.

The 1940's was the decade of the introduction of phsyical treatments, electric convulsive therapy and leucotomy being introduced in the early forties and insulin in 1946, by which year 93% of all hospital admissions were on a voluntary basis. Post war development was rapid and exciting with the establishment of a large research department under the direction of Professor Max Reiss. Barrow reopened in January 1947 and remained under the management of a Visiting Committee of the Local Authority until the National Health Service Management Committee took over on July 5th 1948. Subsequently Barrow and Glenside later the Day Hospital (1951) were run as one unit by the Hospital Management Committee.

There is no time to deal in detail with the more recent history of mental illness in Bristol nor has there been time to deal with other developments in the City such as the pioneering work of Robert Barber in the Bristol Child Guidance Clinic, the development of the subnormality services or the contributions to electro-physiology by the late Professor Gray Walter. Nor have I been able to deal even cursorily with the "opening up" of the mental hospitals which started in the late 1940's nor with the run down of the hospitals which started in about 1956. Maybe some of these topics will find time on another day.

## Figures and Tables

**Fig. 1. f1:**
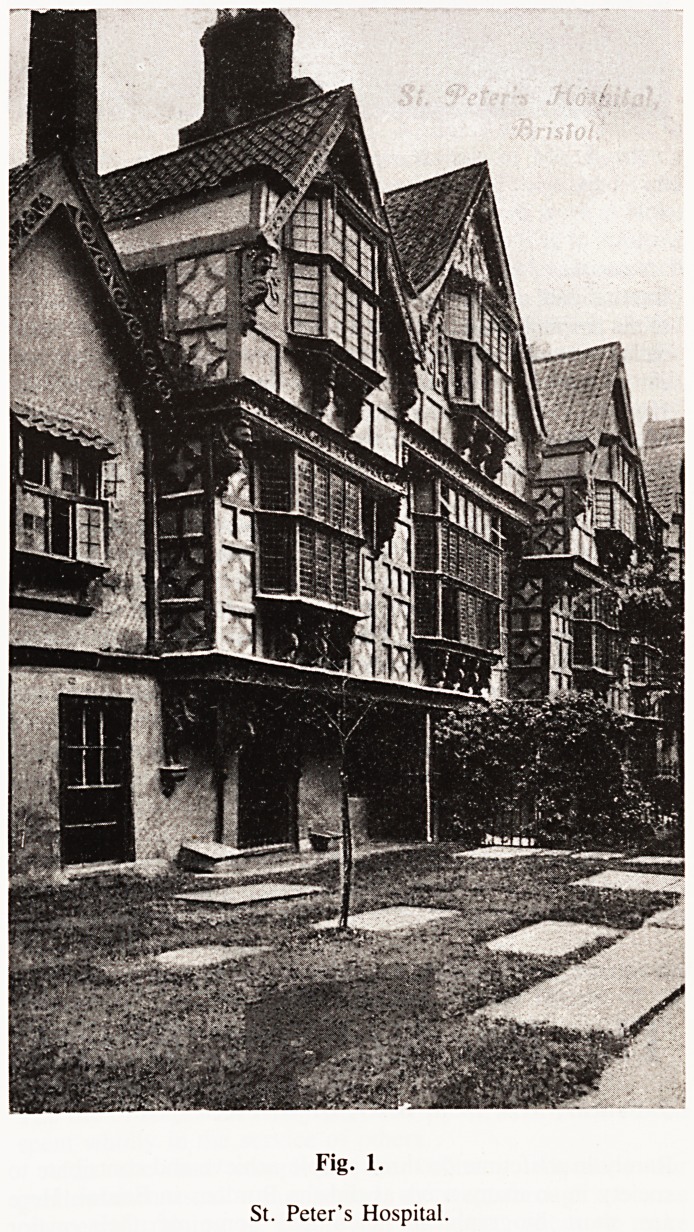


**Fig. 2. f2:**